# Implant-supported orbital prosthesis: a technical innovation of silicone fabrication

**DOI:** 10.1186/s40729-020-00248-0

**Published:** 2020-09-15

**Authors:** Mi Young Eo, Yun Ju Cho, Truc Thi Hoang Nguyen, Mi Hyun Seo, Soung Min Kim

**Affiliations:** 1 Department of Oral and Maxillofacial Surgery, Dental Research Institute, School of Dentistry, Seoul National University, 101 Daehak-ro, Jongno-gu, Seoul, 03080 Korea; 2 Oral and Maxillofacial Microvascular Reconstruction Lab, Ghana Health Service, Brong Ahafo Regional Hospital, P.O. Box 27, Sunyani, Brong Ahafo Ghana

**Keywords:** Dental implant, Silicone orbital prosthesis, Magnet-retained prosthesis, Plastic clay cementation with resin, Three-dimensional (3D) orbital scanning

## Abstract

**Background:**

Silicone-based facial prostheses have traditionally been considered difficult to make and require time-consuming fabrication due to their basic liquid characteristics.

**Methods and results:**

A detailed procedure for creating an ideal silicone orbital prosthesis was developed, including dental implant-supported retention, three-dimensional (3D) orbital scanning with symmetric volume and size measurement based on matching the opposite side, master mold fabrication for convenient pouring of the liquid silicone elastomer, and easy and comfortable management of the prosthesis by the patient.

**Conclusion:**

A silicone orbital prosthesis could be more easily and conveniently produced using updated surgical skills and modern 3D technology. The combination of 3D scanning with digital reconstruction and an innovative fabrication protocol using a reproducible major mold and multiple prototypes fitting resulted in an accuracy personalized facial prosthesis with accessible cost and short production period.

## Background

Currently, the fabrication of facial prostheses usually requires a high level of expertise and long production time. The 3D-printed technique still has several withdraws such as high cost, which is a disadvantage to patients with economic burden, and the limitation of margin refining. Our study aimed to develop an improved protocol that is both time-saving and accessible. The procedure was the combination of 3D facial scanning, digital reconstruction, and innovation of traditional silicone prosthesis production using the reproducible major mold and multiple prototypes. This is a promising protocol in the personalized fabrication of silicone facial prosthesis for diverse facial defect cases.

A 71-year-old woman who had been diagnosed with squamous cell carcinoma of the right frontal sinus was referred to our facility for orbital reconstruction. She had undergone craniofacial fronto-ethmoidectomy with eyeball exenteration by a neurosurgeon and otorhinolaryngologist 16 months previously and had also received induction chemotherapy with docetaxel and cisplatin and then a course of postoperative radiation therapy at a total dosage of 6300 cGy in 28 fractions 14 months previously. Her dura mater had been repaired with a heterologous transplant, and the right temporalis muscle fascia and left forehead glial flap were dissected and covered with duraplasty. A metal plate was placed in the right frontal sinus area, and the patient was scheduled for a routine magnetic resonance imaging (MRI) appointment every 4 months during the first year postoperatively. The defect caused severe functional and esthetic alterations. The direct connection of maxillary sinus and nasal cavity with the environment through a defect at the floor of the orbital made the sinus and nasal mucosa dry and sensitive. It is also important to take into consideration that an intact system of the paranasal cavities is important in swallowing and speech functions. The patient reported that some difficulties in pronunciation, together with the esthetic appearance imperfection, restrained her from participating in social activities.

## Methods (technical advances report)

**Fig. 1:**
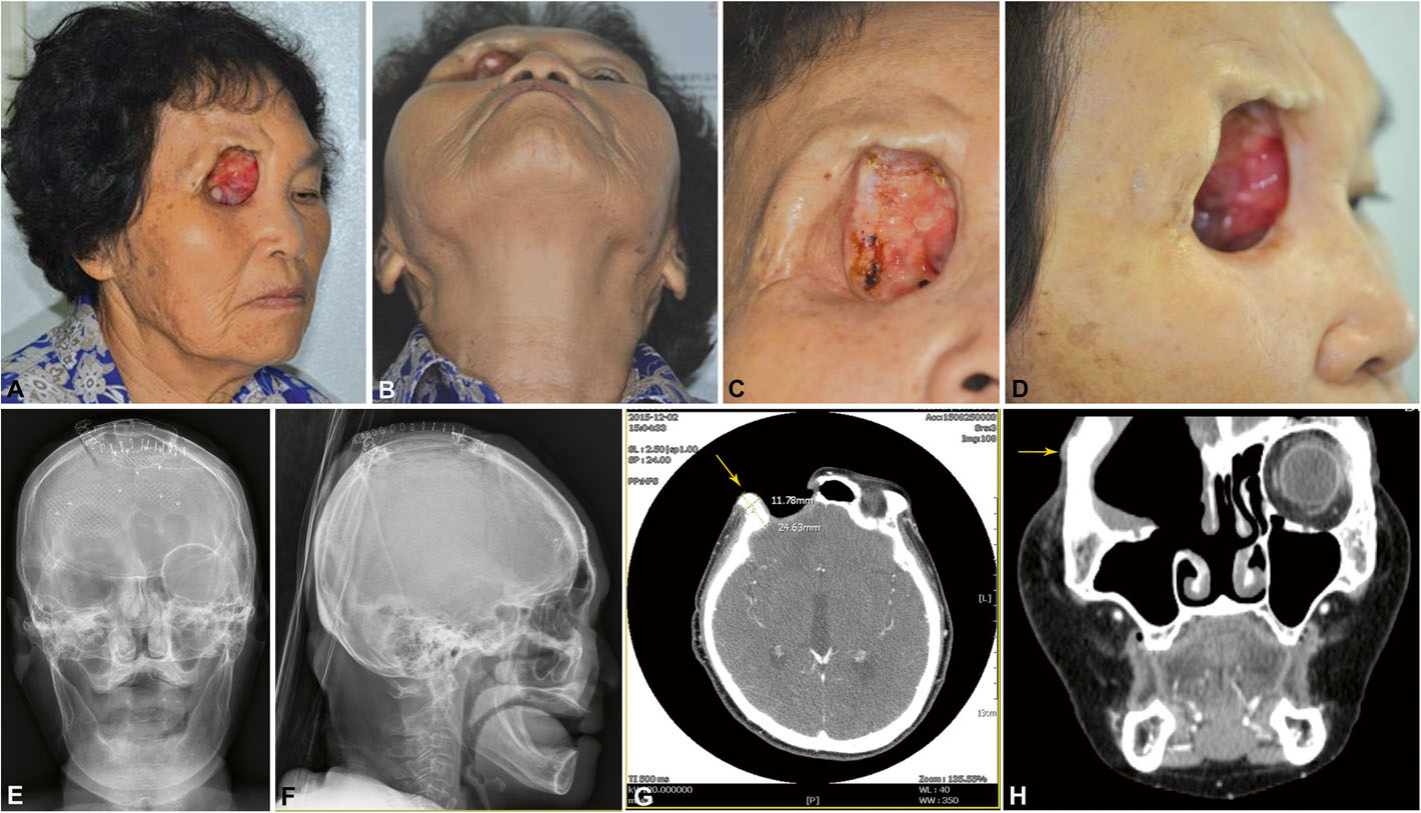
The patient’s clinical and radiographic views after orbital exenteration. The orbital defect on the right side of the face (**a**–**d**), frontal (**e**), and lateral (**f**) skull views, and computed tomography axial (**g**) and coronal (**h**) views are shown along with measurements of residual bony dimensions

At the patient’s first visit to discuss her orbital reconstruction, she communicated that she did not wish to receive any reconstructive surgery or general anesthesia and did not want to be admitted to the hospital. The patient expressed her desire for a natural-looking face (Fig. 1a–d). An orbital prosthesis was recommended instead of microvascular free-flap surgery to meet the patient’s request, and a dental implant that allowed removal of a silicone prosthesis was planned to avoid limitations in her routine MRI examination; any prosthesis that contained a metal frame would have interfered with follow-up scanning. Routine laboratory testing was carried out prior to the dental implant installation procedure, and all results were within the normal range except for mild anemia (hemoglobin, 11.8 g/dl; hematocrit, 34.6%) and an elevated erythrocyte sedimentation rate (34 mm/h).

**Fig. 2:**
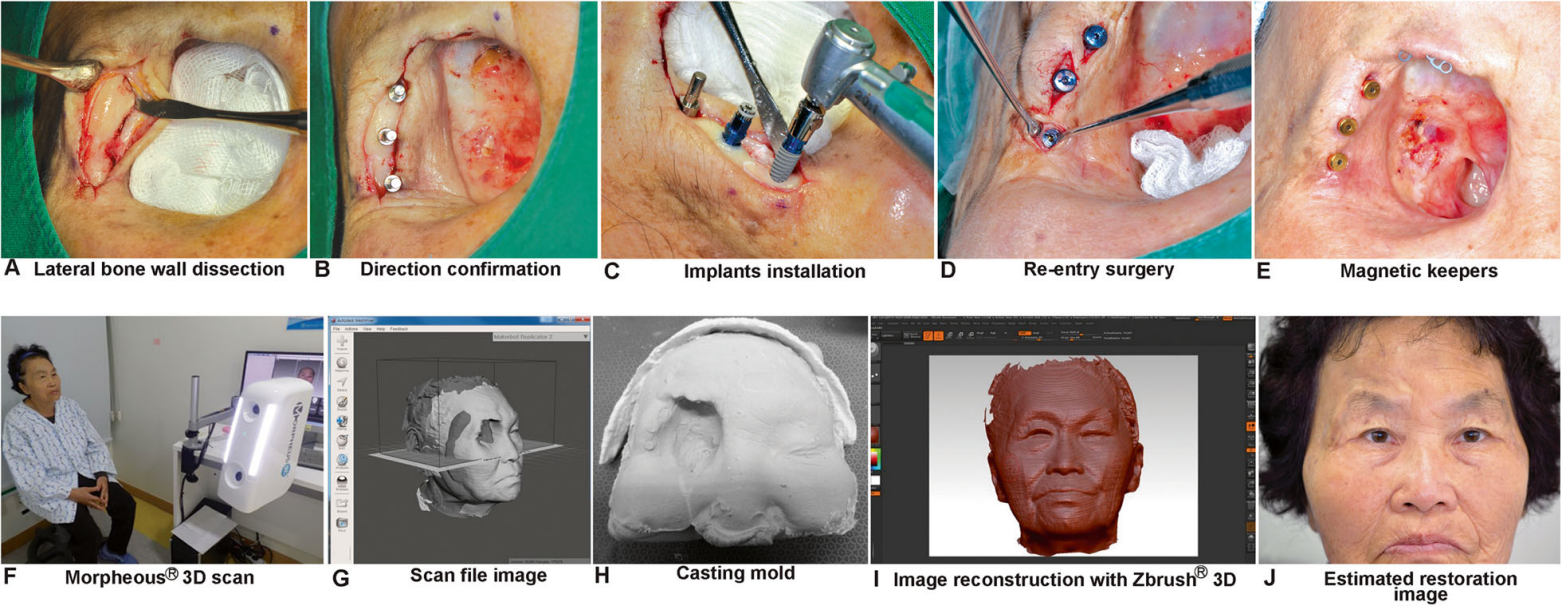
Dental implant installation and 3D scanning with imaging reconstruction. A full-thickness cutaneous flap that exposed the lateral orbital bony wall (**a**); verification of the direction of three implants after drilling using a 2-mm-diameter drill bit (**b**); three Luna® implants (Shinhung Co., Seoul, Korea) 4.0 mm in diameter × 7.0 mm in length were inserted (**c**); the re-entry procedure showing the cover screws being changed into healing abutments 5 months later (**d**); replacement of the healing abutments with the magnetic keeper (MAGFIT IP-B®, Aichi Co., Japan) 4 weeks later (E); 3D facial scanning (**f**) and imaging reconstruction (**g**) with a Morpheus 3D Scanner® (Morpheus Co., Ltd., Seoul, Korea); 3D printing with the data obtained from facial scanning (**h**); 3D molding using ZBrush® image reconstruction software (Pixologic Inc., California, USA) to reconstruct the area with the defect (**i**); and application to the patient’s image (**j**) to design the expected reconstruction

The bony dimensions of the residual orbital wall were studied thoroughly on plain radiographs, which included frontal and lateral skull views (Fig. 2e, f), and computed tomography (CT) scans (Fig. 2g, h) with three-dimensional (3D) images. The treatment plan included placement of three implants in the lateral orbital rim of the zygoma due to the presence of titanium mesh on the superior orbital rim of the frontal bone. With the patient under local anesthesia induced with a lidocaine injection, we placed 4.0-mm-diameter and 7.0-mm-long Luna® implants (Shinhung Co., Seoul, Korea) (Fig. 2a–c). A tapered implant with a suitable length was selected to compensate for the bony wall density and to achieve good primary stability with a > 65 implant stability quotient value from each implant fixture. After inserting the cover screws, suturing was performed in three layers of periosteal membrane overlapping, the subcutaneous layer, and tension-free skin approximation. Six months after implant installation, re-entry surgery was performed under local anesthesia. Healing abutments with an appropriate height were chosen according to the surrounding soft tissue thickness and were applied and tightened with a torque of 25 Ncm, (Fig. 2d). One month later, when the soft tissue had achieved a satisfactory amount of healing and contour, the healing abutments were replaced with 1.0- or 2.0-mm gingival height magnetic keepers (MAGFIT IP system, Aichi Co., Japan) for the removable orbital prosthesis (Fig. 2e). Instead of taking traditional facial impressions to create a master facial stone model, 3D facial scanning using a Morpheus 3D Scanner® (Morpheus Co., Ltd, Seoul, Korea) was performed (Fig. 2f), and facial imaging reconstruction, which included the orbital texture and volume, was carried out (Fig. 2g) for modification of facial master cast fabrication. Formatted reconstruction data were used for 3D molding and printing (Fig. 2h) via image reconstruction ZBrush® software (Pixologic Inc., California, USA) to reconstruct the defect in the right orbital region (Fig. 2i) and finally be applied to the patient’s clinical facial image (Fig. 2j) of the design of the expected reconstruction.

**Fig. 3:**
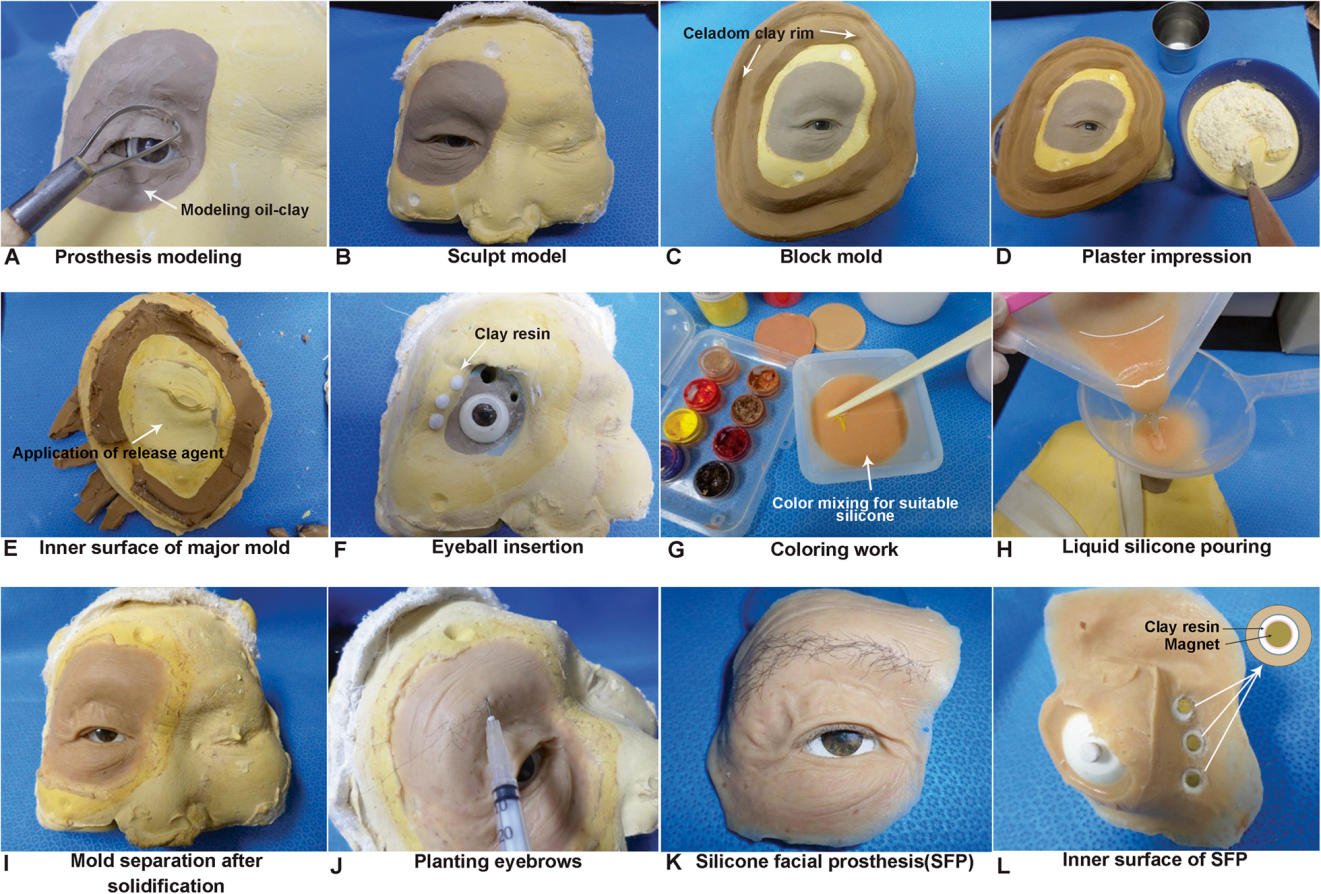
Laboratory fabrication procedures for the orbital prosthesis showing a molding process (**a**, **b**) using a modeling oil-clay (NSP-soft®; Chavant, Inc., New Jersey, USA) on the major facial cast; preparation of a block mold using celadon clay (**c**) to create a mirrored outer appearance of the orbital skin area; pouring the plaster to make the major mold after Vaseline application to the gypsum area excluding the modeling clay (**d**); inner surface of the major mold after solidification (**e**); eyeball attachment after fabrication; the magnet plastic clay (Eyaco®, Goyang, Gyeonggi-do, Korea) mold adaptation on the magnet section in the master cast model (**f**); adding the liquid silicone color component (Ecoflex^TM^ 00-10®; Smooth-On, Inc., Pennsylvania, USA) to the main silicone elastomers (Smooth-Cast®; Smooth-On, Inc., Pennsylvania, USA) to finalize the shade of the silicone base (**g**); pouring the liquid silicone into the main master cast through a small hole in the major mold (**h**); the initial and raw appearance of the silicone orbital prosthesis on the major facial cast (**i**) after solidification of the silicone (at least 6 h); trimming and implanting artificial hair in the eyebrow and eyelash areas (**j**); the outer appearance of the silicone orbital prosthesis (**k**); and the inner surface that contains the posterior part of the eyeball and the individual magnet with firm cementation (**l**)

The laboratory fabrication procedures were also updated and modified by creating a major mold for pouring the liquid silicone and allowing it to set safely. After making a molding orbital prosthesis on the major facial cast using modeling oil clay (NSP-soft®; Chavant, Inc., New Jersey, USA) (Fig. 3a, b), we created a block mold with celadon clay rim (Fig. 3c). After release agent application on the gypsum area of the blood mold, a plaster impression was performed (Fig. 3d). The inner surface of the major mold had a mirror outer appearance of the orbital skin area (Fig. 3e).

The artificial eye was created as a digital image first and then was polished after the determination of the detailed parameters, such as eye size, the correct position of the magnet, skin color, and silicone shade. This artificial eyeball was attached, and the molds of the magnets were made in a uniform shape using clay resin (Eyaco®, Goyang, Korea) and were adapted to the magnet part of the major facial cast (Fig. 3f). The main silicone elastomers (Smooth-Cast®; Smooth-On, Inc., Pennsylvania, USA), dimethyl siloxane polymers, and adhesive primers were used to create an effective bond between the silicone and the substructure [1, 2]. We added a liquid silicone color component (Ecoflex^TM^ 00-10®; Smooth-On, Inc., Pennsylvania, USA) to the main silicone elastomers to produce the basic skin shade based upon our many trial and error laboratory attempts (Fig. 3g). By pouring this liquid silicone base into the assemble combination of the major mold and the major facial cast through a small hole (Fig. 3h), we saved time that would have been used to set the silicone base by editing or handling a silicone surface. After solidification of the silicone base more than 6 h later, the initial raw appearance of the silicone orbital prosthesis on the major facial cast was observed after detaching the major mold (Fig. 3i). Additional trimming and placement of artificial hair in the eyebrow and eyelash areas as well as the outer appearance of the silicone orbital prosthesis was completed (Fig. 3j, k). The magnet molds were attached to the Magnet® (MAGFIT IP system, Aichi Co., Japan) using resin cement and fixed to the silicone base using Loctite® cyanoacrylate instant cement (Henkel Co., USA) in the finalization stage. The inner surface that contained the eyeball and the individual magnet was polished (Fig. 3l).

## Results

The function and esthetic of the patient were improved after the application of facial prosthesis. The retention was achieved with the implant and magnet system. The silicone prosthesis with a well-cover margin also restored nearly normal mastication and swallowing activities. We scored the patient satisfaction to the facial prosthesis as “very poor”, “poor”, “average”, “good”, and “excellent”. The assessed aspects were easiness in applying and removing, no pain or discomfort, stability (during resting and facial muscle movement), chewing and swallowing functions, speech function, and esthetic. All of the assessed criteria achieved “good” and above rating. The patient reported the “excellent” satisfaction for stability and esthetic.

**Fig. 4:**
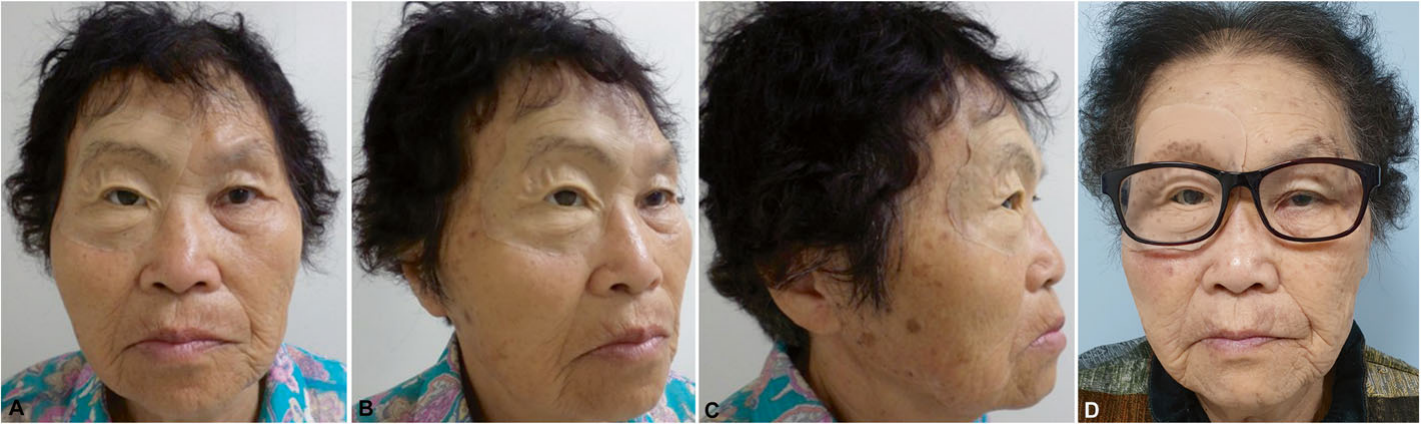
Clinical facial views of the patient with the final silicone orbital prosthesis, frontal (**a**), oblique semi lateral (**b**), lateral (**c**), and eyeglass frontal views (**d**) 4 weeks later

The patient was very happy with the result, and she made her own eyeglass by herself (Fig. 4). The patient and her family were taught how to manage the silicone orbital prosthesis and how to clean the skin surrounding the abutments. We also provided the patient with maintenance guidelines (Table 1) and strongly emphasized the importance of following them. The follow-up schedule was made every 3 months in the first year, and every 6 months in the following years. The patient was also recommended to come to our department anytime an issue appears. During the follow-up visits, the professional maintenance was performed to the implants as well as the silicone prosthesis. The plastic curette and alcohol swab was used for magnet keeper cleansing. The skin and mucosa in the defect region were also cleansed to remove any scalp or accumulated mucus. The silicone prosthesis was meticulously checked by the clinician and the laboratory expert. Any changes in color, silicone margin, or the attachment of the magnet and magnet mold would be quickly recognized and corrected in the same appointment. The patient was re-educated if prosthesis and facial hygiene were not adequately maintained.

**Table 1:** Maintenance care instructions for the patient

**Maintenance care of a silicone face prosthesis** 1. Wipe the front and back surfaces of the prosthesis with lukewarm water. Because the silicone color will lighten over time, wipe the surfaces as carefully as possible. 2. Carefully wipe the prosthesis again with 70% ethanol disinfectant to thoroughly clean the inner surface that contacts the skin directly. 3. When attaching the prosthesis to the face, align the magnets starting from the side with the implant abutments (keeper). After correct alignment with the implant abutments, the prosthesis can be fully attached to the face. 4. After thoroughly cleaning the edge of the prosthesis, partially apply the adhesive with a cotton swab and attach the prosthesis to the skin surface. 5. When removing the prosthesis, carefully remove the magnet side with both hands. 6. Remove any traces of adhesive on the skin and the edges of the prosthesis using the adhesive remover. 7. If any bits of remover component remain, the adhesive strength may decrease during the next use. Therefore, after wiping the prosthesis’s edge thoroughly with remover, clean it again with water and sterilize it with ethanol before storing it. 8. Due to the nature of silicone material, the prosthesis can easily attract dust. Keep the silicone prosthesis in a case after washing, and be careful not to expose it to direct sunlight for extended periods of time	

During the last three and a half years, the patient has been very satisfied with her prosthesis and has experienced no severe misfits or other complications, such as deterioration, unpleasant odor, weakened retention, or discoloration. We planned to re-make the prosthesis every 3 to 5 years due to expected patient skin shade and tonicity changes.

## Discussion

Total removal of the orbital contents was first described as orbital exenteration by Georg Barish in 1583 [3, 4] and is now a common surgical procedure that has several categories depending upon the patient’s malignancy status and the degree of partial excision of the eyelids [5]. Orbital and oculofacial prostheses have been considered the most ideal and viable alternative reconstruction method compared with local flap repositioning or microvascular free-flap coverage [6]. Retention of orbital prosthesis has been achieved using adhesives, attachment to eyeglasses frames or straps, and, more recently, use of dental implants [1, 2, 7]. Three or four implants anchored into the zygomatic or temporal bony orbital rim can be used for reliable outcomes and often allow the patient to avoid radiation or magnetic interferences.

In our current report, the patient underwent postoperative radiotherapy. Even though radiotherapy was originally considered a contraindication for installation of the intraosseous implant due to the risk of osteoradionecrosis (ORN), there are many recent evidence showing that the success rate of dental implant in irradiated bone is comparable to which of the implant in non-irradiated bone [8, 9]. After the evaluation of irradiated dose and the period after radiotherapy (14 months) and the consultant with patient regarding the risk of ORN, the implant installation plan was processed. Under the attentive follow-up, there was no clinical and radiography sign of ORN until the patient’s latest visit.

Several materials have been recommended for orbital prostheses, including acrylic resin, rubber, vinyl plastic, and silicone. Among these biomaterials, silicone is the most adaptable to natural skin colors and textures. We have already addressed one of the weakest points of silicone, which is its inability to be cemented with metal components, using plastic clay resin [1, 2]. Another disadvantage of silicone manipulation was integrated in these updated clinical results. The use of a 3D scanner and printer allowed us to design and edit the exact skin contours and morphologic designs using software, and these changes could be conveniently moved to the final 3D surface mold. In our case, by designing a major mold for easy and retrievable silicone pouring and solidification, we were able to produce several prototypes of silicone base prostheses with differences in silicone margin, eye, and eyebrow design, and color shade of the silicone. The use of reproducible major mold not only results in high accuracy surface morphology of the silicone base but also shortens the fitting stage. All the prototypes were tried on the patient during fitting, and the most suitable one was finalized for the final prosthesis. The combination of 3D scanning with digital reconstruction and an innovative fabrication protocol resulted in an accurate personalized facial prosthesis with accessible cost and short production period.

We developed a procedure for easy and logical fabrication of silicone orbital prostheses. Our suggested method includes dental implant installation on the orbital bony rim, re-entry with a healthy-adapted skin margin, 3D orbital scanning with symmetric volume, and size measurement based on the opposite side of the face, a reproducible major mold fabrication for the convenient pouring of the liquid silicone elastomer, silicone prosthesis setting with an artificial eyeball and plastic clay cementation with silicone surrounding the magnet, and easy adaptation and cleaning by the patient.

## Conclusion

The silicone orbital prosthesis fabricated using the process detailed in this technical advances article can be easily and comfortably used and requires improved surgical skills and innovative 3D-scanning technology.

## Data Availability

The datasets used and/or analyzed during the current study are available from the corresponding author on reasonable request.
